# CT-Defined Osteosarcopenia Is Associated with Mortality in Metastatic Prostate Cancer: A Longitudinal Analysis of Opportunistic CT Imaging

**DOI:** 10.3390/jcm15218453

**Published:** 2026-10-30

**Authors:** Tayler D. Ross, Saleh Tabatabaei, Rose Yakubov, Urban Emmenegger, Cari Whyne, Michael Hardisty

**Affiliations:** 1 Division of Orthopaedic Surgery, University of Toronto, 149 College Street, Toronto, ON M5T 1P5, Canadacwhyne@sri.utoronto.ca (C.W.);; 2 Institute of Medical Science, Temerty Faculty of Medicine, University of Toronto, C. David Naylor Building, 6 Queen’s Park Crescent, Toronto, ON M5S 3H2, Canada; 3 Sunnybrook Research Institute, Sunnybrook Health Sciences Centre, 2075 Bayview Avenue, Toronto, ON M4N 3M5, Canada; s.mirzatabatabaei@mail.utoronto.ca; 4 Institute of Biomedical Engineering, University of Toronto, 164 College Street, Toronto, ON M5S 3G9, Canada; rose.yakubov@mail.utoronto.ca; 5 Temerty School of Medicine, Temerty Faculty of Medicine, University of Toronto, 1 King’s College Circle, Toronto, ON M5S 1A8, Canada; 6 Odette Cancer Centre, Sunnybrook Health Sciences Centre, 2075 Bayview Avenue, Toronto, ON M4N 3M5, Canada

**Keywords:** ageing, artificial intelligence, frailty, prostate cancer, osteosarcopenia, sarcopenia, osteoporosis, musculoskeletal health

## Abstract

**Background/Objectives:** In metastatic prostate cancer, loss of bone density (osteoporosis), muscle quantity and quality (sarcopenia) may contribute to fractures, falls, and mortality. Unfortunately, their longitudinal assessment is poorly characterized. In prostate cancer, ageing, advanced disease, and cancer treatments may accelerate bone and muscle loss. This study assessed longitudinal computed tomography-derived musculoskeletal biomarkers in metastatic prostate cancer and the association between baseline osteosarcopenia and mortality. **Methods:** This retrospective single-centre cohort included 437 patients, 3595 muscle computed tomography (CT) assessments and 2944 bone CT assessments. An automated deep-learning pipeline quantified lumbar trabecular bone mineral density (BMD), psoas muscle volume (PV), and psoas muscle density. Osteosarcopenia was defined as concurrent osteoporosis (BMD < 135 Hounsfield Units (HU)) and sarcopenia (volumetric psoas muscle index (vPMI) < 30). Patient-aware mixed-effects models characterized biomarker trends and reverse-time analysis. Kaplan–Meier analysis and Cox proportional-hazards regressions were utilized to assess mortality. **Results:** Baseline osteosarcopenia affected 75 patients (17.2%). Psoas density decreased by 1.569 HU/year (95% confidence interval (CI) −1.703 to −1.434 HU/yr). Nonlinear models estimated mean declines in psoas volume (−6.42 cm^3^/year) and bone density (−6.01 HU/year). Among deceased patients, modelled final-year declines showed −12.57% in psoas density and −10.28% in psoas volume. Baseline osteosarcopenia was associated with higher all-cause mortality (Hazard Ratio (HR) 1.611; 95% CI 1.134–2.288; *p* = 0.008). **Conclusions:** Osteosarcopenia is common and progressive in metastatic prostate cancer, and muscle loss accelerates near death. Baseline osteosarcopenia independently identifies patients at increased mortality risk. CT imaging provides a scalable approach for longitudinal assessment of musculoskeletal health and offers clinically important information without additional patient burden.

## 1. Introduction

In an ageing population, osteopenia/osteoporosis (low bone mineral density) and sarcopenia (low muscle mass, quality, and strength) are growing public health concerns. In Canada, an estimated 2.3 million individuals are living with osteoporosis [1]. While it is challenging to identify sarcopenia prevalence due to variation in measurement techniques, estimates are between 9.9 and 40.4% of the population over 60 years old [2,3]. The coexistence of osteoporosis and sarcopenia is called osteosarcopenia [4]. In the Canadian Longitudinal Study on Ageing, 22.3% of 8888 older adults met a definition of osteosarcopenia. This was associated with increased odds of falls and fracture among men [5].

Prostate cancer is the 4th most common cancer in Canada, with one in eight men affected, and it is Canada’s 3rd leading cause of cancer death [1,6]. The incidence and prevalence of prostate cancer are increasing with our ageing population. Unlike many malignancies, survival in prostate cancer is high, with 91% of patients diagnosed surviving at least 5 years [7]. Patients with metastatic prostate cancer are particularly susceptible to deteriorating musculoskeletal health because of advanced age, malignancy-associated changes, and cancer-directed therapies. Androgen deprivation therapy (ADT) is a cornerstone therapy in both metastatic and non-metastatic prostate cancer, with ~75% of prostate cancer patients receiving ADT treatment [8]. ADT targets prostate cancer progression by decreasing circulating androgens, primarily testosterone, which is crucial for both bone and muscle maintenance in men [9]. The impacts of ADT on musculoskeletal health are significant, including an increase in fracture and falls risk over time [10,11,12,13,14]. In prostate cancer, the rate of osteoporosis is over 80% after >10 years of ADT [15]; sarcopenia impacts over half of prostate cancer patients (56.6%; 21.8–87.4%) [2,3].

Bone mineral density (BMD) is conventionally evaluated with dual-energy X-ray absorptiometry (DEXA). Sarcopenia assessments are primarily limited to clinical strength and physical performance testing, as little standardization of imaging-based techniques exists to diagnose sarcopenia [16,17,18]. Routine cancer surveillance CT scans offer an opportunity to assess musculoskeletal biomarkers without additional imaging. CT-derived measures can be used to assess bone density via lumbar vertebral trabecular attenuation in Hounsfield units (HU) and muscle quantity and quality through psoas volume and psoas muscle attenuation [19,20]. Lower CT muscle attenuation has been associated with a higher level of myosteatosis (fatty infiltration) [21]. A systematic review and meta-analysis of 40 cancer studies (21,222 patients) associated muscular fatty infiltration with greater mortality (pooled Hazard Ratio (HR) 1.75, 95% CI 1.60–1.92) [22]. In prostate cancer, CT-based bone and muscle measurements have been associated with survival-related outcomes in localized disease, and lower muscle attenuation was related to shorter survival in castration-resistant disease [19,20].

Although prior literature supports the clinical relevance of CT imaging-based biomarkers, their longitudinal progression in metastatic prostate cancer remains poorly characterized, particularly near the end of life. The primary objective of this study was to characterize changes in osteoporosis (bone density) and sarcopenia (psoas volume and density) related imaging biomarkers using opportunistic cancer surveillance CT scans. The secondary objective was to assess the relationship between baseline osteosarcopenia and overall survival. An exploratory reverse-time analysis characterized biomarker trajectories prior to death. We hypothesized that bone and muscle biomarkers would decline over the disease course and that baseline osteosarcopenia would be associated with shorter survival.

## 2. Materials and Methods

### 2.1. Study Design

This retrospective single-centre observational cohort used opportunistic disease-surveillance CT Imaging at the Odette Cancer Centre, Sunnybrook Health Sciences Centre (Toronto, ON, Canada). Institutional ethics approval was obtained at Sunnybrook Health Sciences Centre (REB#1801).

### 2.2. Data Collection

The study utilized CT scans collected from the Ontario Clinical Imaging Network (OCINet), from patients seen at the Odette Cancer Centre, Sunnybrook Health Sciences Centre, Toronto, ON, Canada, between 1 January 2009 and 30 December 2022. Follow-up imaging was captured until 30 August 2024.

- Inclusion criteria: patients with metastatic prostate cancer considered for systemic therapy between 1 January 2009 and 30 December 2022, with CT imaging including the lumbar spine.
- Exclusion criteria: no relevant CT imaging; non-metastatic prostate cancer; missing patient height for sarcopenia assessment.

Clinical data were captured from electronic medical health records until 30 June 2024, and compiled into a Research Electronic Data Capture (REDCap) database. Patient data collected included: standard demographic data (including height and weight), Gleason score/grade, prostate-specific antigen results, tumour, nodes and metastasis staging, age at initial prostate cancer diagnosis, age at first available CT scan, and treatment information.

### 2.3. Automated Image Analysis Pipeline

CT scans involving the lumbar spine (digital imaging and communications in medicine format) were processed using an automated image analysis pipeline built using 3D Slicer (version 5.6), including custom Python-based (version 3.9.13) analysis and deep-learning models [23,24]. Briefly, the pipeline identifies lumbar vertebrae (L1–L5) and their centroids and segments the vertebral bodies using a custom convolutional neural network (UNet) [25,26]. The segmented region is eroded to remove the cortical shell and enable measurement of the vertebral body trabecular bone mineral density (BMD). The psoas muscle is segmented using TotalSegmentator, an existing nnU-NET segmentation model [27]. The psoas segmentation is cropped to a consistent region using the L2/3 and L4/5 boundary planes, defined as perpendicular to the vector connecting the L2-L3 and L4-L5 vertebral body centroids. This ensures consistent scan-to-scan segmentation of the psoas muscle. Comparison of ground-truth manual segmentations of the psoas muscle with TotalSegmentator demonstrated a consistent under-segmentation of the psoas volume; therefore, a correction factor (x1.19) was applied to derive the true psoas volume. Based on the segmented regions of interest, the following biomarkers were calculated: average lumbar vertebral body trabecular BMD from L1 to L5 (mean and standard deviation), psoas muscle volume (PV) (of the cropped region) and psoas muscle density (mean and standard deviation). Volumes were quantified in cm^3^ and densities in Hounsfield units (HU).

### 2.4. Identification of Metastatic Involvement

Spinal metastatic lesions are common in prostate cancer. These lesions are typically osteoblastic and lead to hyperdense regions of bone. Given that, identification of radiographically involved metastatic vertebrae was required to identify changes in bone density reflective of local osteoblastic lesions vs. systemic bone health. To automate identification of non-metastatically involved vertebral levels, we developed a random forest classifier utilizing information from radiology reports (extracted with LLaMA 3.1 (8B)), BMD biomarkers, and a custom convolutional model trained to classify metastatically involved vertebrae. In an internal validation using a held-out test set, the classifier achieved an accuracy of 85.0%, sensitivity of 84.6%, specificity of 85.1%, positive predictive value of 69.4%, and negative predictive value of 93.3%. Where questions remained regarding metastatic involvement, manual review of the imaging and radiology report was performed, including review of the patient’s next CT scan to determine if the previous ambiguous lesion became clearly involved. Metastatically involved vertebrae were excluded from systemic BMD calculations. Note: occult or sub-radiographic metastatic involvement could not be fully excluded.

### 2.5. Definitions: Osteoporosis, Sarcopenia, and Osteosarcopenia

Baseline CT-defined osteoporosis was defined as average vertebral trabecular BMD < 135 HU in metastasis-free vertebral bodies [28]. To define sarcopenia, we used a three-dimensional (3D) volumetric psoas muscle index (vPMI), defined as PV (cm^3^) divided by height^3^ (m^3^). This 3D metric was found to have a strong correlation (r^2^ = 0.91) to the existing two-dimensional (2D) psoas muscle index [24]. Based on the 2D psoas muscle index threshold of <6.35 cm^2^/m^2^ for diagnosing sarcopenia, a vPMI < 30 was used as the threshold for sarcopenia. Osteosarcopenia represents the concurrence of osteoporosis and sarcopenia, reflecting patients with an average BMD < 135 HU and vPMI < 30.

### 2.6. Statistical Analysis

Statistical analysis was performed using RStudio (version 4.4.1). Continuous variables were summarized as medians and interquartile ranges; categorical variables as counts and percentages. Baseline phenotype groups were compared using omnibus Kruskal–Wallis tests for continuous variables, and Fisher-Freeman-Halton Monte Carlo tests for categorical distributions. Holm adjustment was applied to exploratory pairwise comparisons where applicable.

Longitudinal changes in bone density and muscle biomarkers were assessed using linear mixed-effects models to incorporate all eligible CT scans. Time was measured in years from patients’ first CT scan, with patient-specific random intercepts and slopes accounting for repeated measurements. Linear, quadratic, and cubic trajectories were compared using maximum likelihood, with selected models refitted using restricted maximum likelihood. Population-level trajectories and 95% confidence intervals (CI) were estimated.

For reverse-time analyses, CT measurements were aligned to each patient’s date of death and normalized to the first eligible CT. All eligible examinations within 10 years before death were included. Nonlinear muscle trajectories were evaluated using generalized additive mixed-effects models, while bone density was assessed using a linear mixed-effects model.

Overall survival was calculated from the first CT scan until death or last documented follow-up. All death dates were retained and validated. Kaplan–Meier curves and log-rank tests compared survival between baseline phenotypes. Multivariable Cox proportional-hazards regression assessed the association between baseline osteosarcopenia and mortality, adjusting for age at first CT, time from diagnosis, log-transformed prostate-specific antigen, and Gleason risk group. Missing covariates were excluded using complete-case analysis. Proportional hazards were evaluated using Schoenfeld residuals, with alternative model specifications examined in sensitivity analysis. Statistical significance was defined as a two-sided *p* < 0.05.

## 3. Results

### 3.1. Study and Patient Characteristics

**Figure 1:**
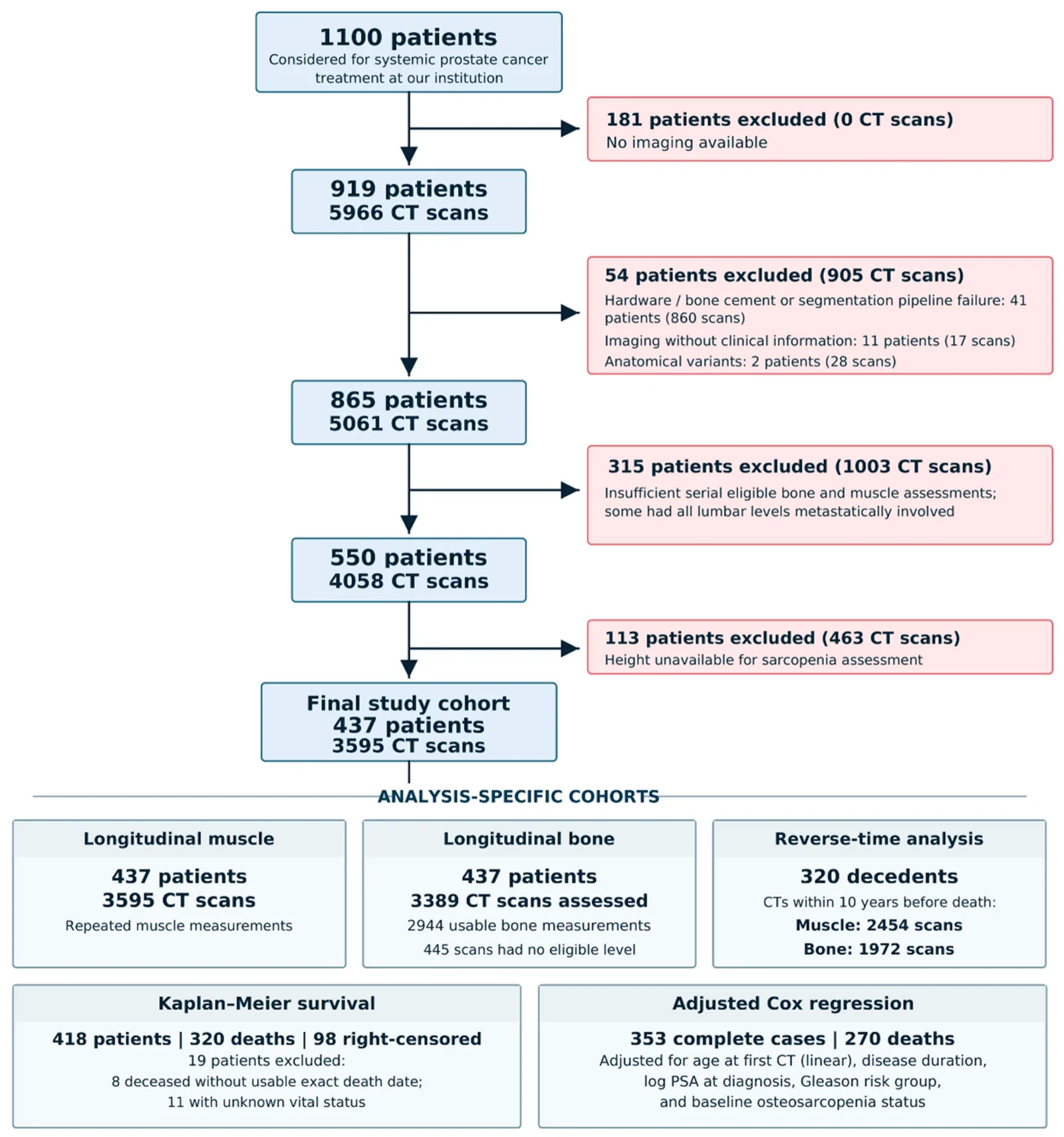
Study selection and analysis cohorts: Flowchart illustrating patient selection, exclusion criteria, and the final cohorts included in longitudinal imaging and survival analyses.

Overall, 437 patients with 3595 CT scans met inclusion criteria. See Figure 1 for details of patient exclusion. Patient characteristics categorized by the total cohort and baseline phenotypes are summarized in Table 1. At first eligible CT, 180 patients (41.2%) had no osteoporosis or CT-derived sarcopenia (NOS). CT-derived sarcopenia alone was present in 130 patients (29.7%), and 52 patients (11.9%) had osteoporosis alone. Osteosarcopenia was found in 75 patients (17.2%). Therefore, 127/437 (29.1%) met osteoporosis criteria, and 205/437 (46.9%) met sarcopenia criteria at the first CT scan. Median age at diagnosis was 66.6 years (IQR 61.0–72.5), and median age at first CT was 72.3 years (IQR 66.3–78.4). Gleason scores were categorized into risk groups. Low Risk relates to a Gleason score equal to or less than 6 (128/437; 29.3%. Medium Risk is a Gleason score of 7 (57/437; 13.0%), and high risk is a Gleason score of 8–10 (218/437; 49.9%). Gleason Score and risk group were unknown in 34/437 patients (7.8%). There was no statistical difference in Gleason risk group between phenotypes. Mortality status was known for 426/437 (97.5%) of patients, with 328/437 (75.1%) deceased, with no difference in distribution (deceased, alive, unknown) between the 4 baseline groups.

### 3.2. Baseline Phenotype and Overall Survival

**Figure 2:**
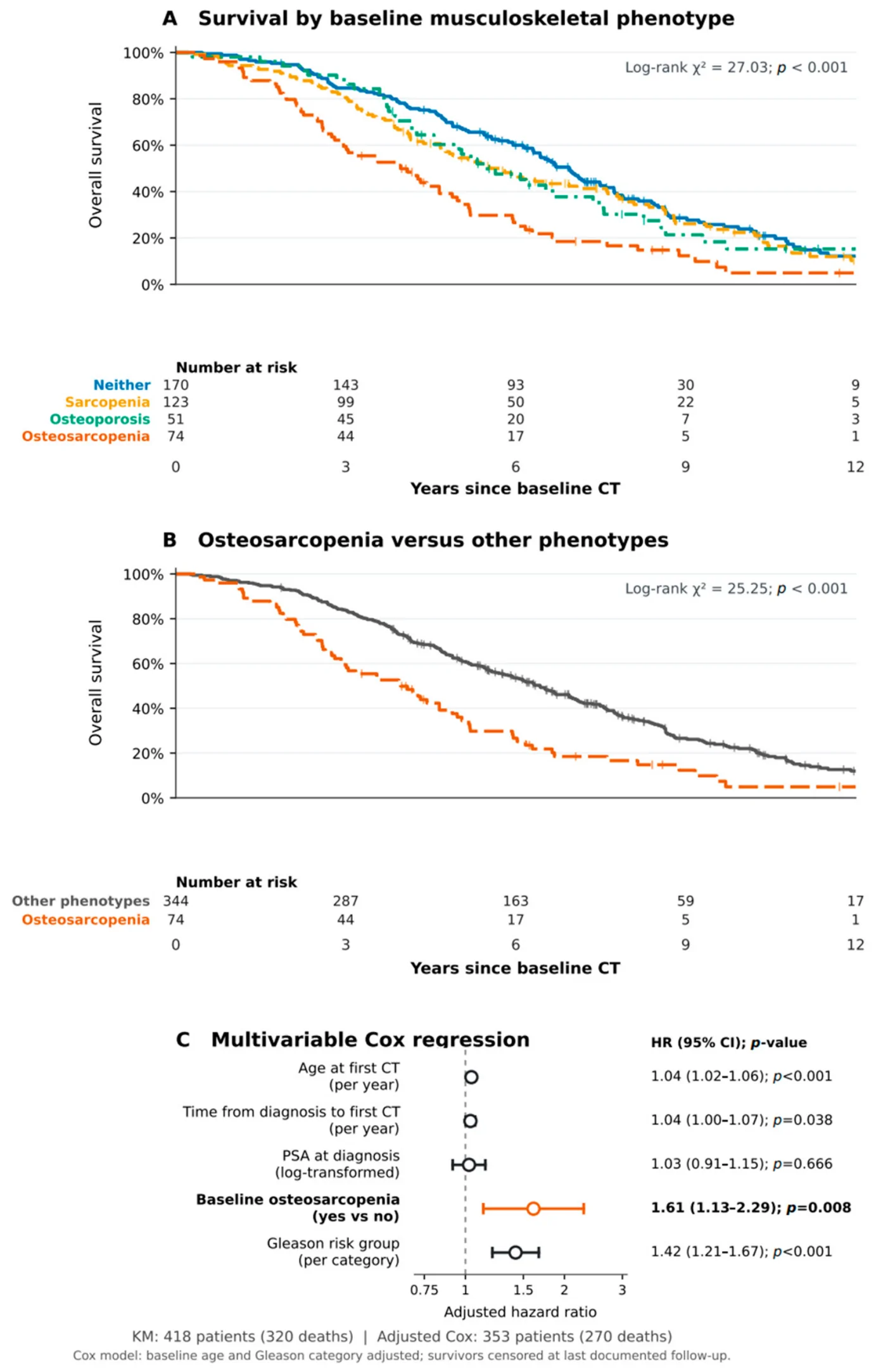
Association between baseline osteosarcopenia and overall survival. (**A**) Kaplan–Meier survival curves stratified by four baseline musculoskeletal phenotypes. (**B**) Kaplan–Meier comparison of patients with and without osteosarcopenia. (**C**) Multivariable Cox regression showing adjusted hazard ratios with 95% confidence intervals. Numbers at risk are displayed beneath the survival curves.

The evaluable Kaplan–Meier cohort included 418 patients (320 deaths and 98 right-censored individuals): 170 NOS patients, 123 with CT-derived sarcopenia alone, 51 with osteoporosis alone, and 74 with osteosarcopenia. Survival differed among the four phenotypes (log-rank χ^2^ = 27.03, *p* < 0.001; Figure 2A) and between the baseline osteosarcopenia cohort and all others combined (log-rank χ^2^ = 25.25, *p* < 0.001; Figure 2B).

Unfortunately, of the 437 patients, 19 had unknown or missing vital status, and another 65 were missing at least one covariate, and the remaining multivariable Cox cohort comprised 353 complete cases with 270 deaths. After adjustment for age at first CT, time from diagnosis to first CT, PSA at diagnosis, and Gleason Risk Group, osteosarcopenia was associated with increased all-cause mortality (HR 1.611, 95% CI 1.134–2.288; *p* = 0.008; Figure 2C). Age at first CT (HR 1.043/year; 95% CI 1.024–1.062; *p* < 0.001), time from diagnosis to first CT (HR 1.036/year; 95% CI 1.002–1.071; *p* = 0.038), Gleason risk group (HR 1.420/category increase; 95% CI 1.207–1.671; *p* < 0.001) were associated with mortality. Log-PSA at diagnosis was not associated (HR 1.025, 95% CI 0.915–1.150; *p* = 0.666). Adjusted effect estimates are shown in Figure 2.

### 3.3. Bone and Muscle Biomarkers over Time

**Figure 3:**
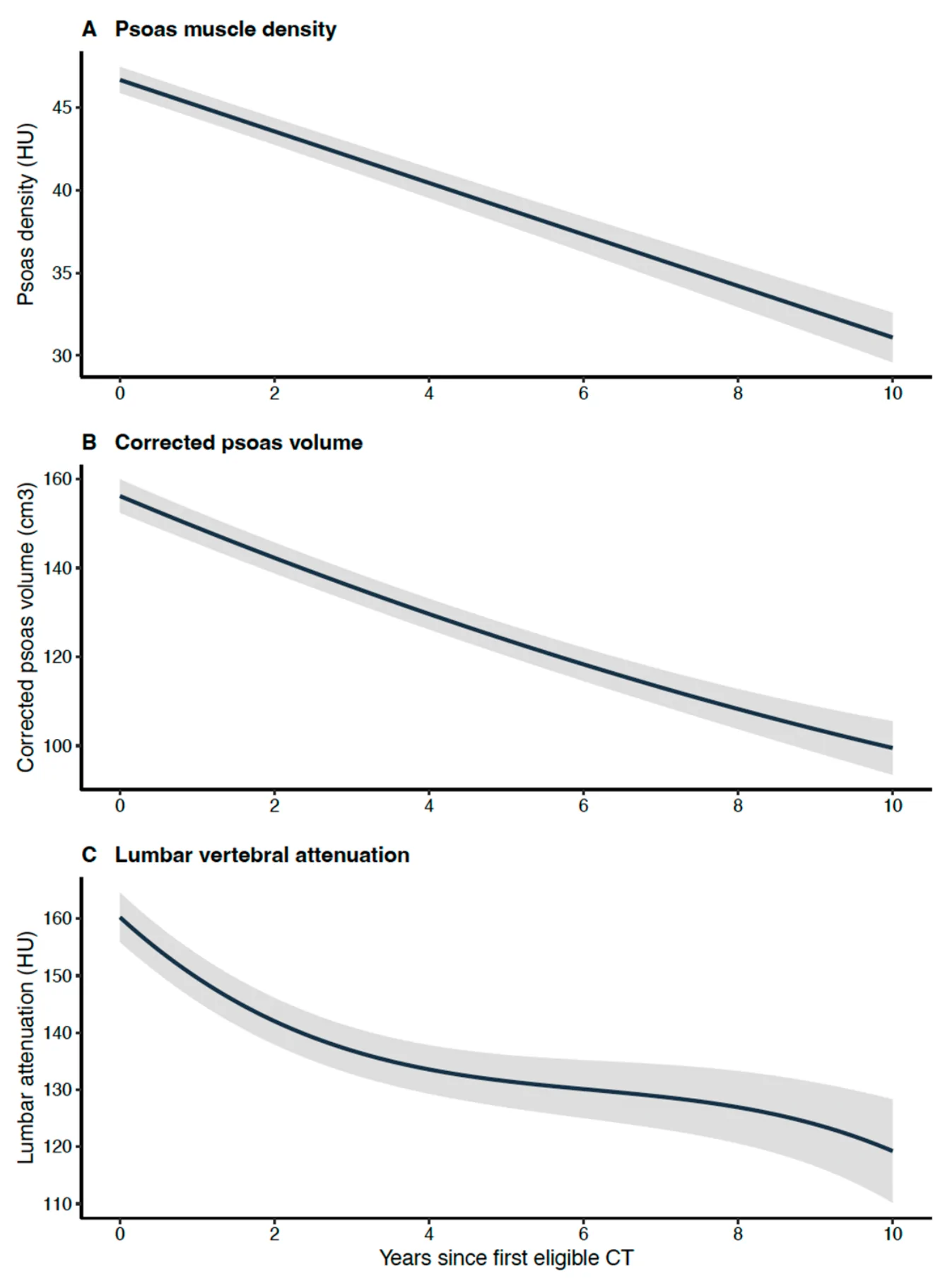
Longitudinal changes in musculoskeletal biomarkers: Population-level mixed-effects trajectories of (**A**) psoas muscle attenuation, (**B**) corrected psoas muscle volume, and (**C**) bone density over time from the first CT examination. Shaded areas represent 95% confidence intervals.

All 437 patients contributed 3595 CT scans for muscle analysis. Psoas muscle density was modelled using random-intercept-random-slope and found to decrease by 1.569 HU/year (95% CI 1.434–1.703 HU/year decline; *p* < 0.001). Corrected psoas muscle volume followed a quadratic trajectory (intercept 155.89 cm^3^, linear term −6.90 cm^3^/year, quadratic term 0.096 cm^3^/year^2^; overall time, *p* < 0.001); fitted values corresponded to a 32.09 cm^3^ decrease over the first 5 years. Bone density followed a cubic mixed-effects trajectory (intercept 159.62 HU; linear −10.06 HU/year, quadratic 0.9251 HU/year^2^, cubic −0.023 HU/year^3^; overall time *χ*^2^ = 346.67, 3 df; *p* < 0.001). The fitted five-year decline was 30.05 or 6.01 HU/year. These results are visualized in Figure 3. At each patient’s final available CT—not a standardized follow-up time—236/437 (54.0%) met the osteoporosis threshold and 362/437 (82.8%) met the sarcopenia threshold, and 199/437 (45.5%) met both thresholds to be classified as osteosarcopenic.

### 3.4. Phenotype-Specific Longitudinal Comparisons

Formal phenotype over time interactions showed no evidence that the rate of psoas density decline differed across groups (χ^2^ = 4.98, 3 df; *p* = 0.173). Corrected psoas volume showed phenotype-specific linear time terms (χ^2^ = 42.36, 3 df; *p* < 0.001); however, additional phenotype-specific quadratic terms did not improve fit (*p* = 0.196). Bone density trajectories differed by phenotype in the linear (*p* < 0.001) and quadratic (*p* < 0.001) time components, but not in cubic interactions (*p* = 0.597). Group definitions use the baseline biomarker values; therefore, regression to the mean and floor effects complicate specific interpretation.

### 3.5. Reverse-Time Analysis of Musculoskeletal Biomarkers Before Death

**Figure 4:**
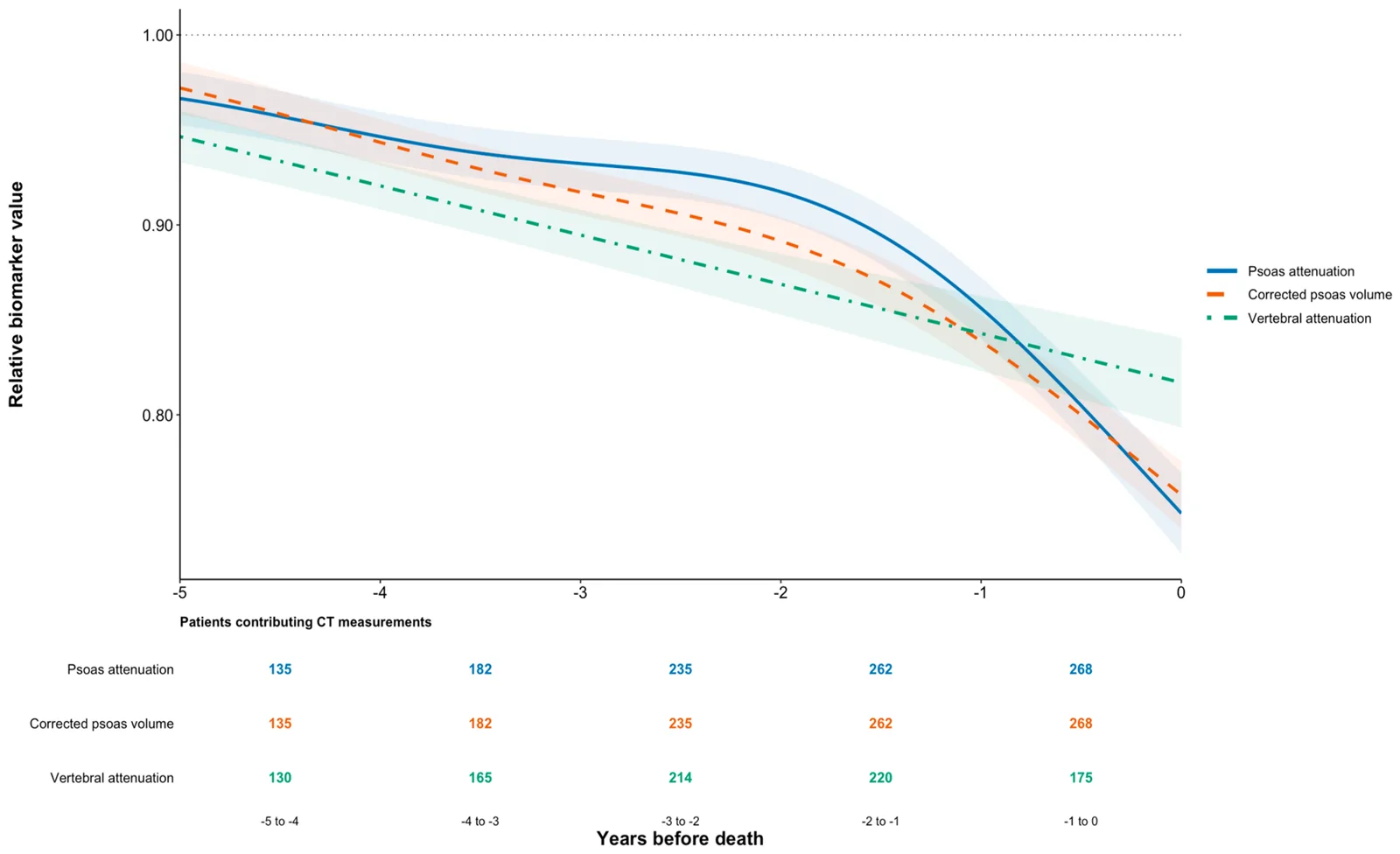
Musculoskeletal changes preceding death: Reverse-time trajectories of psoas muscle attenuation, corrected psoas muscle volume, and bone density during the final five years of life. Values are normalized to the first eligible CT measurement. Shaded areas represent 95% confidence intervals. The number of contributing patients is shown for each annual interval. Relative biomarker values represent each CT measurement normalized to the patient’s first eligible CT measurement (baseline = 1.0), with values below 1.0 indicating a proportional decline from baseline (e.g., 0.8 represents a 20% reduction).

Among 320 patients with known death dates, the final 10 years before death included 2454 muscle and 1972 eligible bone-density CT scans. Generalized additive mixed-effects models supported nonlinear terminal changes for psoas density and corrected psoas muscle volume. Fitted decreases during the final year of life were −12.57% in psoas density and −10.28% in corrected psoas volume. Relative bone density maintained an approximately linear relationship near death (−0.026 relative units/year, 95% CI −0.031 to −0.021; *p* < 0.001). Figure 4 displays the final five years of the reverse-time trajectories on a normalized scale so relative changes in biomarkers can be compared directly. For muscle measures, 135 patients contributed 5 years prior to death and 268 in the year preceding death. Bone density contributions were 130 patients 5 years prior to death and 175 in the year preceding death. Fewer bone density assessments were available due to lumbar metastatic involvement.

## 4. Discussion

In prostate cancer, disease surveillance CT scans represent an opportunity to quantify bone and muscle health and identify osteosarcopenia without additional cost to the patient or healthcare system [17,29,30]. In this longitudinal study, opportunistic surveillance CT allowed recurring evaluation of bone density, psoas density, and psoas volume. Using all eligible serial CT scans in patient-aware mixed-effects models, we observed a linear decline in psoas muscle attenuation and nonlinear declines in corrected psoas volume and bone density. Baseline osteosarcopenia was associated with increased all-cause mortality after adjustment for selected clinical variables. Mean psoas density decreased by approximately 1.57 HU/year (3.29%/year). Corrected psoas volume and bone density declined in non-linear manners. Over the first five years, the models estimate reductions of 32.09 cm^3^ and 30.05 HU, respectively, representing roughly 6.42 cm^3^/year and 6.01 HU/year. While these are non-linear relationships, this approximates to −3.29%, −4.11%, and −3.82% changes in psoas density, volume, and bone density.

We were unable to show that any of the phenotypes had differing rates of psoas density changes. Psoas volume and BMD did show phenotype-specific time components. However, baseline classification uses the same biomarkers modelled over follow-up; therefore, regression to the mean, floor effects and mathematical coupling limit the ability to interpret these differences.

At baseline, CT-defined sarcopenia was identified in 46.9% of patients and osteosarcopenia in 17.2%. These estimates should not be interchanged with estimates based upon functionally assessed sarcopenia. Myosteatosis provides an imaging-based assessment of muscle quality, as lower CT muscle attenuation correlates with intramuscular fat content and has been associated with worse outcomes in oncology populations [21,22]. In prostate cancer, McDonald et al. associated CT bone and muscle measures with noncancer mortality in non-metastatic cohorts [19]. Whereas Lee et al. reported a survival association for muscle attenuation in castration-resistant disease [20]. These studies support the relevance of CT biomarkers in our metastatic cohort.

In our survival analysis, osteosarcopenia was associated with higher all-cause mortality (adjusted HR 1.611, 95% CI 1.134–2.288; *p* = 0.008). This estimate remains after adjustment for the recorded covariates, although residual confounding by systemic therapy, timing and duration of ADT, bone-targeting agents, and tumour burden remains plausible. Similarly, Wang et al. highlight the impact of osteosarcopenia on survival in cancer patients [4], where individuals with osteosarcopenia had worse disease-free survival (HR 2.17; 1.62–2.92) [4]. The association between osteosarcopenia and mortality represents a key finding in this study.

Reverse-time analysis suggests that psoas density and psoas volumes experience a terminal deterioration during the final year of life. This is similar to other reports of accelerated muscle loss prior to death in cancer patients [31,32]. Babic et al. showed that pancreatic cancer patients experiencing the greatest muscle loss had decreased survival [33]. In our study, patients experienced psoas density and volume decreases during the final year of −12.57% and −10.28%, respectively. In contrast, bone density maintained a relatively linear decline. This result is striking and necessitates further study to evaluate its prognostic role.

It is estimated that 3.1–5.6% of community-dwelling adults and 18.9–33.1% of elderly populations are sarcopenic [34]. These estimates are lower than our observed sarcopenia rate (46.9%) at first imaging, suggesting an impact of prostate cancer and/or its treatment on muscle health. This mirrors results from Kovač et al., who reported sarcopenia in 43.8% of patients across 24 prostate cancer studies [3]. Surov and Wienke reviewed the prevalence of sarcopenia in patients with solid tumours from 280 studies (81,814 patients), estimating sarcopenia prevalence of 48.5–55.4% in early prostate cancer and 72.2–79.9% in palliative settings [2]. The latter aligns with a sarcopenia prevalence of 82.8% at the final scan in our analysis.

This study does have limitations. This was a retrospective single-centre study of metastatic prostate cancer patients with irregular follow-up, variable imaging protocols, and possible informative dropout. Restriction to patients with suitable imaging and clinical data may have introduced selection bias. Occult metastatic involvement may influence imaging biomarkers. Our imaging-only approach to sarcopenia lacks a strength or physical performance validating test. Also, psoas measurements are not necessarily reflective of systemic muscle mass. Lastly, residual confounding may exist from systemic therapies, duration of ADT, tumour burden, nutrition, and bone-targeted agents. The threshold for defining sarcopenia based on vPMI, while based on correlation to the established two-dimensional psoas muscle index, has not been validated in patients with metastatic prostate cancer. Alternate vPMI thresholds may impact the association between baseline osteosarcopenia and mortality.

The automated nature of the imaging pipeline is particularly useful in diseases such as prostate cancer where routine disease surveillance imaging is performed. Conventional assessment of osteoporosis and sarcopenia requires dedicated investigations that are inconsistently performed. In contrast, opportunistic analysis of surveillance CT scans allows for repeated assessment of musculoskeletal health without added demand on the patient or the healthcare system. Future studies should integrate standardized CT acquisition, functional assessments, and treatment histories to prospectively evaluate clinical utility.

## 5. Conclusions

Serial opportunistic CT scans demonstrated longitudinal musculoskeletal deterioration in bone density, psoas density, and psoas volume. The declines in muscle quality and quantity accelerated near death, and patients became more osteosarcopenic during their disease course. Baseline CT-defined osteosarcopenia was associated with higher adjusted all-cause mortality. Automated extraction of musculoskeletal biomarkers from routine cancer surveillance CT imaging provides a scalable approach for longitudinal assessment of musculoskeletal health and offers clinically important information without additional patient burden.

## Figures and Tables

**Table 1:** Baseline patient characteristics by CT-defined musculoskeletal phenotype.

Characteristic	Overall (n = 437)	Neither (n = 180)	Sarcopenia (n = 130)	Osteoporosis (n = 52)	Osteosarcopenia (n = 75)	*p*
**Clinical and disease characteristics**						
Age at prostate cancer diagnosis, years	66.6 (61.0–72.5)	65.0 (59.2–69.6)	66.4 (60.8–71.7)	67.2 (62.6–71.7)	71.8 (66.3–76.6)	<0.001
Age at first CT, years	72.3 (66.3–78.4)	68.7 (63.5–74.9)	71.4 (65.4–78.8)	72.4 (67.0–76.1)	78.6 (74.2–84.3)	<0.001
Diagnosis to first CT, years	3.9 (0.6–8.2)	3.1 (0.4–6.7)	3.3 (0.5–8.9)	4.1 (1.2–7.9)	5.5 (2.7–10.2)	0.003
PSA at diagnosis, ng/mL	15.1 (8.0–35.9)	15.3 (7.9–32.0)	15.0 (7.7–39.9)	11.9 (7.6–25.6)	18.8 (10.8–40.0)	0.240
**Histopathology**						**0.021**
Adenocarcinoma	405 (92.7%)	169 (93.9%)	121 (93.1%)	50 (96.2%)	65 (86.7%)	
Other	5 (1.1%)	2 (1.1%)	1 (0.8%)	2 (3.8%)	0 (0.0%)	
Unknown	27 (6.2%)	9 (5.0%)	8 (6.2%)	0 (0.0%)	10 (13.3%)	
**Gleason score group**						**0.084**
≤6	128 (29.3%)	43 (23.9%)	46 (35.4%)	16 (30.8%)	23 (30.7%)	
7	57 (13.0%)	33 (18.3%)	11 (8.5%)	4 (7.7%)	9 (12.0%)	
8–10	218 (49.9%)	93 (51.7%)	65 (50.0%)	27 (51.9%)	33 (44.0%)	
Unknown (code 11)	34 (7.8%)	11 (6.1%)	8 (6.2%)	5 (9.6%)	10 (13.3%)	
**Baseline CT-derived musculoskeletal biomarkers**						
Psoas muscle attenuation, HU	47.6 (42.2–53.1)	49.7 (44.6–55.7)	48.6 (43.0–52.5)	44.8 (40.1–51.5)	42.0 (36.9–48.2)	—
Corrected psoas volume, cm^3^	156.3 (128.3–182.6)	178.9 (159.4–198.8)	130.6 (116.8–147.2)	180.4 (165.5–196.8)	121.4 (101.3–144.2)	—
Volumetric psoas muscle index	30.4 (25.9–35.6)	35.5 (32.7–39.2)	26.2 (23.5–28.4)	34.9 (31.7–37.7)	23.9 (21.7–27.5)	—
Lumbar trabecular attenuation, HU	157.5 (129.7–189.1)	177.1 (158.3–208.4)	165.8 (150.4–191.4)	117.6 (107.7–127.7)	110.6 (97.8–124.2)	—
**Follow-up status (descriptive)**						
**Vital status**						**0.277**
Recorded deceased	328 (75.1%)	129 (71.7%)	95 (73.1%)	40 (76.9%)	64 (85.3%)	
Recorded alive	98 (22.4%)	46 (25.6%)	30 (23.1%)	12 (23.1%)	10 (13.3%)	
Unknown	11 (2.5%)	5 (2.8%)	5 (3.8%)	0 (0.0%)	1 (1.3%)	
Age at death, years	78.7 (72.4–84.6)	76.4 (70.0–82.5)	78.6 (71.5–84.3)	78.7 (73.4–81.4)	84.6 (78.3–88.4)	<0.001

Baseline patient characteristics by musculoskeletal phenotype: Values are presented as median (interquartile range) or n (%). Between-group comparisons were performed using Kruskal–Wallis tests for continuous variables and Fisher–Freeman–Halton exact tests with Monte Carlo simulation for categorical variables. CT, computed tomography; HU, Hounsfield units; PSA, prostate-specific antigen; vPMI, volumetric psoas muscle index.

## Data Availability

The data presented in this study are available from the corresponding author upon reasonable request. The data are not publicly available due to patient privacy and ethical restrictions.

## Abbreviations

The following abbreviations are used in this manuscript:

ADT	Androgen deprivation therapy
BMD	Bone mineral density
CI	Confidence interval
CT	Computed tomography
DEXA	Dual-energy X-ray absorptiometry
HR	Hazard ratio
HU	Hounsfield units
IQR	Interquartile range
NOS	No osteoporosis or sarcopenia
OCINet	Ontario Clinical Imaging Network
PSA	Prostate-specific antigen
PV	Psoas muscle volume
REDCap	Research Electronic Data Capture
vPMI	Volumetric psoas muscle index
